# Long-Term Patency of Plastic Stents in Elderly Patients with Common Bile Duct Stones: A Prospective Pilot Study †

**DOI:** 10.3390/jcm14165715

**Published:** 2025-08-12

**Authors:** Han Taek Jeong, Gwang Hyo Yim, Jimin Han

**Affiliations:** Department of Internal Medicine, Daegu Catholic University School of Medicine, Daegu 42472, Republic of Korea; htjeong@cu.ac.kr (H.T.J.); 98im@naver.com (G.H.Y.)

**Keywords:** choledocholithiasis, endoscopic retrograde cholangiopancreatography, stents, prospective studies, aged

## Abstract

**Background/Objectives**: Endoscopic retrograde cholangiopancreatography (ERCP) with stone extraction is the standard treatment for common bile duct (CBD) stones. However, when complete removal is not feasible, the temporary placement of a plastic stent (PS) is commonly used. This study aimed to assess 12-month stent patency in elderly patients with CBD stones. **Methods**: This prospective study included patients aged 70 years or older who underwent ERCP with PS placement for CBD stones at Daegu Catholic University Medical Center from March to December 2023. Patients were followed every three months with laboratory tests and abdominal radiographs. Stent dysfunction was defined as either cholangitis or cholecystitis due to stent migration or occlusion. **Results:** Of 12 enrolled patients, 2 were lost to follow-up. The remaining 10 had a median age of 85 years. The median stone diameter and number were 16.5 mm and 3, respectively. Two patients (20%) experienced stent dysfunction at 1.4 and 2.7 months and underwent successful stent exchange. Of the remaining eight, one declined further ERCP, while seven underwent elective ERCP at 12 months. Among them, five achieved successful stone removal and two required stent exchange. Kaplan–Meier analysis showed 90% patency at 1.4 months and 80% at 2.7 months, maintained through 12 months. **Conclusions**: Plastic stents showed an acceptable 12-month patency in most elderly patients. Early complications suggest the need for close monitoring during the first three months, after which long-term stent maintenance may be feasible in selected cases. Larger studies are needed to validate these findings.

## 1. Introduction

Common bile duct (CBD) stones are typically managed with endoscopic retrograde cholangiopancreatography (ERCP) [1]. However, approximately 10% to 15% of cases are considered difficult due to stone characteristics or anatomical variations [2]. In such situations, temporary biliary drainage using plastic stent (PS) placement is often employed as an alternative strategy [1,2].

Temporary stenting has been shown to reduce stone size and facilitate delayed removal at follow-up ERCP, typically within a few months of initial drainage, with or without the addition of ursodeoxycholic acid (UDCA) [3,4]. However, when complete clearance remains unachievable despite repeated attempts, long-term biliary stenting with regular exchange may be required. According to the European Society of Gastrointestinal Endoscopy (ESGE) guideline, PSs placed for incomplete CBD stone clearance should be exchanged within 3–6 months to prevent infectious complications such as cholangitis [1].

ERCP is associated with inherent procedural risks, which are amplified with repeated procedures [5]. A meta-analysis showed octogenarians had higher rates of mortality compared with younger patients, whereas nonagenarians had increased rates of bleeding, cardiopulmonary events, and death [6]. Furthermore, when ERCP-related complications occur, they are often associated with worse clinical outcomes and longer hospital stays in elderly patients [7]. In this context, extending the stent exchange interval to 6 to 12 months may help reduce the cumulative procedural burden in selected elderly or high-risk patients. Several studies have reported acceptable long-term stent patency with on-demand exchange beyond 6 or even 12 months [8,9,10], whereas a randomized trial demonstrated that scheduled 3-month exchanges significantly reduced the incidence of cholangitis [11]. However, most of the available data are from retrospective or heterogeneous cohorts, and prospective studies evaluating long-term stent patency, particularly in elderly patients, remain scarce.

This study therefore aimed to evaluate the 12-month patency and clinical outcomes of PS in elderly patients with incompletely cleared CBD stones.

This article is a revised and expanded version of an abstract entitled ‘Long-term patency of plastic stents in elderly patients with common bile duct stones: A prospective pilot study’, which was presented at the International Pancreatobiliary Meeting (IPBM) 2025, Seoul, Korea, in April 2025 [12].

## 2. Materials and Methods

### 2.1. Study Design and Population

This prospective pilot study was conducted at Daegu Catholic University Medical Center. Although the first procedure among eligible patients was performed in March 2023 as part of routine clinical care, patients were considered enrolled in the study only after providing written informed consent during their first outpatient follow-up visit, in accordance with the IRB-approved protocol. Therefore, the actual enrollment period was from April 2023 to December 2023, and patients were followed for up to 12 months after enrollment, with the final follow-up completed in December 2024. Patients who underwent ERCP with PS placement for CBD stones were considered for inclusion. Eligible patients were adults aged 70 years or older, with CBD stones confirmed by imaging, and in whom stone extraction was not attempted during the index ERCP. Stone removal was not attempted in patients with large or multiple common bile duct stones in combination with either significant comorbidities or antiplatelet therapy.

Patients were excluded if they were unable to attend regular outpatient follow-up for at least six months, had biliary obstruction due to malignancy such as cholangiocarcinoma or pancreatic cancer, or had benign strictures related to conditions such as primary sclerosing cholangitis or chronic pancreatitis. Patients who underwent infundibulotomy during stent placement were also excluded.

All patients received a bile acid combination therapy consisting of UDCA and chenodeoxycholic acid (CDCA) (CnU, Daewoong Pharmaceutical Co., Ltd., Seoul, Korea), administered orally at a dose of one tablet three times daily throughout the follow-up period, as part of the study protocol.

The study protocol was reviewed and approved by the Institutional Review Board (IRB) of Daegu Catholic University Medical Center (IRB number: 2023-03-005). Written informed consent for study participation, including prospective follow-up and bile acid therapy, was obtained after the procedure but prior to study enrollment. Transportation costs were reimbursed using research funds in accordance with the approved study protocol.

### 2.2. Procedure

All procedures were performed using a side-viewing duodenoscope (JF-260V or TJF-260V, Olympus, Tokyo, Japan) under conscious sedation with intravenous midazolam and pethidine. In cases where patients had large or multiple CBD stones in combination with either significant comorbidities or were receiving antiplatelet therapy, the endoscopist determined that complete stone extraction posed a high procedural risk or was technically infeasible [1]. In such cases, endoscopic sphincterotomy was intentionally omitted, and a 7 Fr, 5 cm double pigtail plastic stent (Advanix™; Boston Scientific, Natick, MA, USA) was placed for biliary drainage. After the procedure, patients were monitored for immediate post-procedural complications and were observed for one to two days. They were discharged once clinically stable.

### 2.3. Outcome Measurement

Baseline data, including demographics, comorbidities, use of antithrombotic agents, laboratory findings, and imaging results, were collected at the time of enrollment. Patients were followed at 3-month intervals with blood tests, including complete blood count (CBC) and liver function tests, as well as abdominal radiographs to assess stent position and function. The primary outcome was stent patency at 12 months, which was defined as the continued presence of a functioning stent without clinical evidence of dysfunction. Stent dysfunction was defined as the occurrence of acute cholangitis or acute cholecystitis resulting from stent occlusion or migration. The diagnostic criteria for acute cholangitis and cholecystitis were based on the Tokyo Guidelines [13]. The secondary outcome was the occurrence of ERCP-related adverse events, including post-ERCP pancreatitis (PEP), bleeding, and perforation.

### 2.4. Statistical Analysis

The initial target sample size was 80 patients, based on an expected 6-month stent patency rate of 95%, as estimated from the previous literature and institutional experience. Using a 95% confidence level, a 5% margin of error, and an assumed 10% dropout rate, the required sample size was calculated accordingly. However, due to challenges in patient enrollment, particularly because patients with large or multiple CBD stones who were considered unsuitable for removal were less common than initially expected, only 12 patients were ultimately included during the study period. In light of this limitation, the study was redefined and reported as a prospective pilot study to explore the feasibility and describe the outcomes of long-term plastic stenting.

Descriptive statistics were used to summarize baseline characteristics and outcomes. Continuous variables were presented as medians with interquartile ranges (IQRs), and categorical variables as frequencies with percentages. Kaplan–Meier analysis was performed to estimate stent patency over time. Statistical analyses were conducted using IBM SPSS Statistics for Windows, version 26.0 (IBM Corp., Armonk, NY, USA).

## 3. Results

### 3.1. Study Flow and Baseline Characteristics

**Figure 1:**
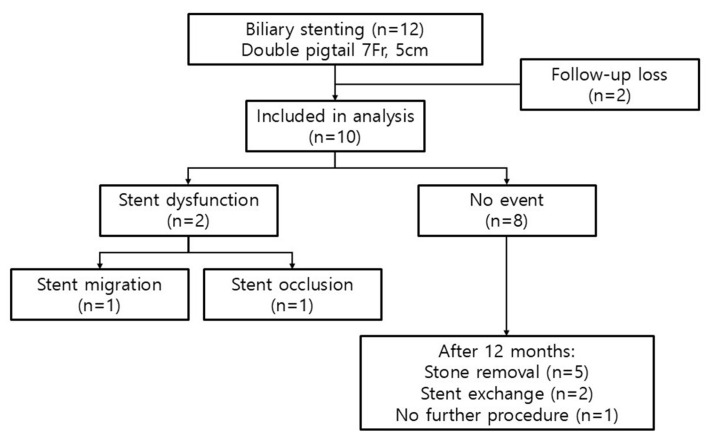
Patient flow and clinic al outcomes. Of 12 patients who received biliary stenting, 10 were included in the final analysis. Two experienced early stent dysfunctions, and eight had no events. At 12 months, five underwent stone removal, two had elective stent exchange, and one was managed with observation.

A total of 12 patients underwent biliary plastic stenting with double pigtail PS (7 Fr, 5 cm). Two patients were lost to follow-up, resulting in a final analysis cohort of 10 patients (dropout rate: 16.7%) (Figure 1). Baseline characteristics are summarized in Table 1. The median age was 85 years (IQR 79–86), with five male patients (50%). The median body mass index (BMI) was 24.4 kg/m^2^ (IQR 20.8–26.2). Hypertension was the most common comorbidity (60%), followed by diabetes mellitus (40%), ischemic heart disease (40%), and cerebrovascular accident (30%). Most patients (80%) were taking aspirin, and one patient (10%) was on clopidogrel. The median diameter of the largest common bile duct stone was 16.5 mm (IQR 11.9–21.4), and the median number of stones was three (IQR 2–4). Laboratory values at baseline showed a median white blood cell count of 14,000/μL, aspartate aminotransferase 81 U/L, alanine aminotransferase 81 U/L, total bilirubin 2.5 mg/dL, alkaline phosphatase 170 U/L, and gamma-glutamyl transferase 270 U/L.

### 3.2. Stent-Related Events and Patency

**Figure 2:**
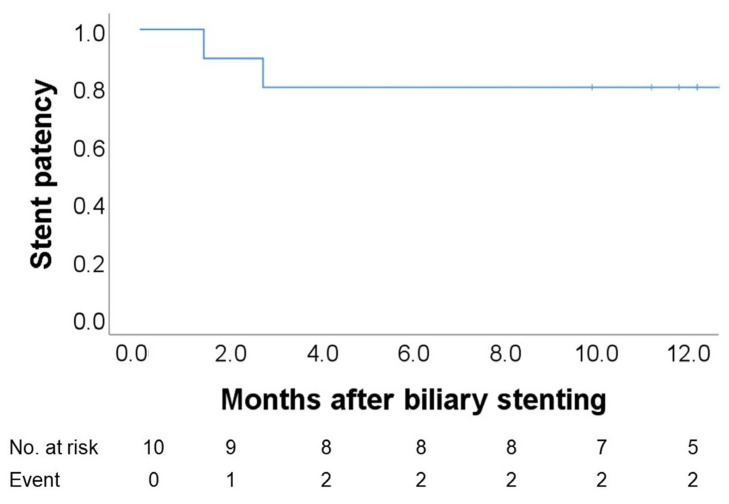
Kaplan–Meier curve of 12-month stent patency. Stent patency was 90% at 1.4 months and 80% at 2.7 months after stent placement, with no additional events observed during the remainder of the 12-month follow-up.

Over a median follow-up of 9.4 months, two patients experienced stent-related complications. One patient developed cholangitis due to stent migration at 1.4 months after the initial ERCP, while another developed cholangitis due to stent occlusion at 2.7 months. Both underwent successful stent exchange without further adverse events. Kaplan–Meier analysis of stent patency showed a patency rate of 90.0% at 1.4 months and 80.0% at 2.7 months, which remained unchanged through 12 months (Figure 2). It showed that all adverse events occurred within the first 3 months, and the patency remained stable thereafter, with no dysfunctions observed between 3 and 12 months.

### 3.3. Clinical Course and Adverse Events

The clinical course during the follow-up period is shown in Figure 1. Of the ten patients included in the final analysis, two experienced stent dysfunctions, while the remaining eight showed no evidence of stent dysfunction. At 12 months, one of the eight patients declined to undergo further ERCP and was instead followed conservatively through outpatient monitoring, with plans for intervention only if symptoms developed. The remaining seven patients underwent elective ERCP for stent exchange. Among them, five showed significant stone fragmentation or size reduction, allowing for successful stone removal. No ERCP-related adverse events such as PEP, cholangitis, bleeding, or perforation were observed during the follow-up period. Detailed individual data, including baseline characteristics, stent-related events, and 12-months outcomes, are presented in Table 2. Of the seven patients who underwent follow-up ERCP, five achieved complete stone removal, while two underwent stent exchange due to incomplete clearance.

## 4. Discussion

Current ESGE guidelines recommend that PS be exchanged within 3–6 months to minimize the risk of infectious complications [1]. However, strict adherence to this schedule may not always be feasible in frail or elderly patients, as the risks associated with repeated ERCP procedures, including bleeding, cardiopulmonary complications, and mortality, are significantly higher in individuals aged 80 years and older [6].

In this study, we evaluated the safety and feasibility of maintaining PSs in elderly patients with difficult CBD stones, particularly when stone removal was deemed high-risk due to comorbidities or general condition. Although patient selection was based on clinical judgment regarding overall status, a formal frailty assessment was not performed, which may have limited the objectivity of risk stratification in this elderly cohort.

This pilot study demonstrated a stent patency rate of 80.0% at 2.7 months, which was maintained throughout 12 months. These findings are consistent with previous studies reporting 6-month patency rates between 88.0% and 94.0%, and 12-month rates ranging from 75.4% to 79.0% [8,10,14]. Notably, all stent-related complications in this study occurred within the first three months after plastic stent placement, suggesting that this may represent a high-risk period for dysfunction.

The mechanism of PS occlusion involves early bacterial colonization and biofilm formation, leading to luminal narrowing and irregular stent surfaces that promote biliary sludge accumulation [15]. This process begins within 4 to 8 weeks after placement and forms the theoretical basis for recommending stent exchange every three months [16]. In this study, only one patient experienced early occlusion, and no additional events occurred thereafter. Although the small sample size limits generalizability, our findings suggest that stent occlusion tends to occur during an early high-risk period, and that once this window passes, the progression of biofilm and sludge accumulation may slow, resulting in a plateau phase with stable long-term patency.

Double pigtail stents are generally considered less prone to migration than straight stents due to their anchoring loops, whereas straight stents align with the bile duct axis and are more susceptible to distal migration [17,18]. Duodenal perforation, although rare, has been more frequently reported with straight stents and is thought to result from such migration [17]. For these reasons, a double pigtail stent was used in all patients in this study. Only one case of early migration was observed, while all other stents remained in place throughout the follow-up period. This pattern is hypothesized to reflect similar dynamics to occlusion, in which early intraductal pressure elevation due to biofilm or sludge may predispose to migration but a more stable state is subsequently achieved.

Among the eight patients with stents in place at 12 months, seven consented to ERCP. Five of these showed significant stone size reduction with fragmentation, allowing for successful stone clearance. Several studies have demonstrated that delayed stone removal after plastic stenting can be effective [4,19]. One study reported that patients receiving UDCA in addition to stenting had higher stone clearance rates at 3 months compared to stenting alone (93.8% vs. 84.4%) [4]. In our cohort, all patients received a combination of UDCA and CDCA, and five of seven patients achieved successful stone removal. These findings support the viability of prolonged stenting followed by interval stone extraction in selected cases.

Based on these observations, we propose a risk-adapted follow-up strategy. Rather than performing routine 3-month exchanges in all patients, clinicians could consider intensive monitoring during the first 1 to 2 months, such as monthly laboratory tests and imaging. If no signs of stent dysfunction are observed during this period, the stent duration may be safely extended to 6 to 12 months before elective exchange or stone removal. This approach may help reduce procedural burden while maintaining safety in appropriately selected patients.

## 5. Limitations

This study has several limitations. First, although originally designed with a target sample size of 80 patients, only 12 were ultimately enrolled due to recruitment challenges. As such, the study was conducted and interpreted as a pilot. Second, the small sample size limits the ability to draw definitive conclusions. Due to the limited sample size, multivariate analysis to adjust for potential confounding variables could not be performed. Third, as a single-center study, the findings may not be generalizable to other settings. Lastly, several study protocol-related factors may have limited the comprehensiveness of data collection and interpretation. For example, follow-up imaging relied solely on plain radiographs, which may have underestimated subtle stent dysfunction. Hepatotoxicity monitoring and evaluation of potential drug interactions between bile acid therapy and antiplatelet use were not conducted. Additionally, microbiological analysis of occluded stents was not performed, limiting insight into infection-related mechanisms.

Importantly, the absence of a control group, such as patients undergoing routine stent exchange, precludes direct comparison and weakens the ability to draw firm conclusions regarding the safety and feasibility of long-term stent retention. However, it is worth noting that the follow-up strategy used in this study reflects real-world clinical practice, which may enhance the applicability of our findings to similar patient populations.

## 6. Conclusions

In conclusion, this pilot study suggests that long-term stent patency may be achievable in selected patients, particularly when early stent-related complications are absent. A follow-up strategy emphasizing early intensive monitoring followed by individualized stent management may represent a safe and cost-effective alternative to routine short-interval exchanges. Larger, multicenter prospective studies are warranted to confirm these findings and further refine risk-based follow-up protocols.

## Figures and Tables

**Table 1:** Baseline characteristics of patients who underwent ERCP.

Variables	Total (*n*= 10)
Age, yrs	85 (79–86)
Male	5 (50.0)
BMI, kg/m^2^	24.4 (20.8–26.2)
Comorbidity	
HTN	6 (60.0)
DM	4 (40.0)
IHD	4 (40.0)
CVA	3 (30.0)
Medication	
Aspirin	8 (80.0)
Clopidogrel	1 (10.0)
Stone size, mm	16.5 (11.9–21.4)
Stone number	3 (2–4)
Laboratory tests before ERCP	
WBC, μL	14,000 (10,850–19,325)
AST, U/L	81 (31–139)
ALT, U/L	81 (28–155)
Total bilirubin, mg/dL	2.5 (1.4–5.0)
ALP, U/L	170 (111–316)
GGT, U/L	270 (180–314)

Data are presented as number (%) or median (interquartile range). BMI, body mass index; ERCP, endoscopic retrograde cholangiopancreatography; HTN, hypertension; DM, diabetes mellitus; IHD, ischemic heart disease; CVA, cerebrovascular accident; WBC, white blood cell; AST, aspartate aminotransferase; ALT, alanine aminotransferase; ALP, alkaline phosphatase; GGT, gamma-glutamyl transferase.

**Table 2:** Individual patient outcomes following plastic biliary stenting.

Variables	No 1	No 2	No 3	No 4	No 5	No 6	No 7	No 8	No 9	No 10
Age, yrs	85	84	89	86	72	86	78	85	83	70
Sex	Male	Female	Male	Female	Female	Female	Male	Male	Female	Male
Antithrombotic use	Dual	None	Aspirin	Aspirin	Aspirin	None	Aspirin	Aspirin	Aspirin	Aspirin
Number of stones (Baseline)	3	2	2	7	4	5	1	3	2	1
Largest stone size, mm (Baseline)	10.2	27.2	10.0	18.1	12.0	14.9	28.1	11.9	20.3	21.8
Stent dysfunction	Migration	None	None	Cholangitis	None	None	None	None	None	None
ERCP at 12 months	NA	Yes	Yes	NA	Yes	No	Yes	Yes	Yes	Yes
Number of stones (At 12 months)	NA	Fragmented	1	NA	Fragmented	NA	1	1	2	3
Largest stone size, mm (At 12 months)	NA	12.2	10.0	NA	7.0	NA	27.8	9.5	20.5	18.1
Final clinical outcome	NA	SSR	SSR	NA	SSR	NA	SE	SSR	SE	SSR

ERCP, endoscopic retrograde cholangiopancreatography; NA, not available; SSR, successful stone removal; SE, stent exchange.

## Data Availability

The data presented in this study are available on request from the corresponding author due to privacy restrictions.

## Abbreviations

The following abbreviations are used in this manuscript:

ERCP	Endoscopic retrograde cholangiopancreatography
ESGE	European Society of Gastrointestinal Endoscopy
CBD	Common bile duct
PS	Plastic stent
UDCA	Ursodeoxycholic acid
CDCA	Chenodeoxycholic acid
CBD	Complete blood count
PEP	Post-ERCP pancreatitis
SSR	Successful stone removal
SE	Stent exchange
